# Teaching machines to optimizing machining parameters: using independent fuzzy logic controller and image data

**DOI:** 10.1007/s42452-022-04987-0

**Published:** 2022-03-16

**Authors:** Harshavardhan Mamledesai, Yufan Zheng, Rafiq Ahmad

**Affiliations:** Laboratory of Intelligent Manufacturing, Design, and Automation (LIMDA), Department of Mechanical Engineering, NW University of Alberta, 9211 116 street, Edmonton, Canada

**Keywords:** Tool condition monitoring, Parameter optimization, Vision systems, CNN, Fuzzy logic

## Abstract

Optimization of machining parameters like cutting speed, feed, and depth of cut is one of the extensively studied fields in the past two decades. While researchers agree optimization of these parameters is essential, there is no conscience as to what the objective of the optimization should be. The studies consider production cost, production time, surface finish, among others, as the objective of parameter optimization, but there are very few studies that consider the manufacturer prescribed tool life as the criteria for parament optimization. Among the methods that do consider tool life as an optimization objective, very few are closed-loop systems and these systems are facing challenges to generalizing when the application changes or the machining material changes or the tool geometry changes. Considering this, a novel image feedback using a convolution neural network-based method combined with principles of fuzzy logic is used to optimize machining parameters. Since the system is based on online feedback from the images of the inserts, it can be used for different materials, and the system is invariant to the different tool geometries and grades as the decisions are based on the wear mechanisms detected. The hybrid system is validated through experimentation for the turning application, but the methodology can be easily adapted for other machining applications.

## Introduction

**Fig. 1:**
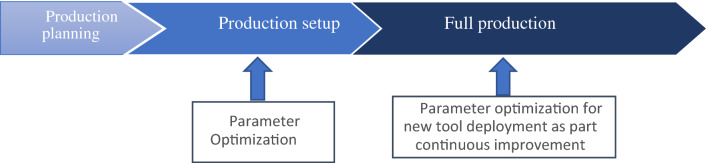
Sequence of production evolution and the parameter optimization interventions in different steps

In the past decades, the optimization of machining parameters like cutting speed, feed, and depth of cut are extensively studied [1]. The studies are warranted as these parameters affect different facets of machining, which include but not limited to surface finish, load on the machine, power consumption, tool life [2–4]. Usually in a mass manufacturing setup, these parameters are optimized at the initial stages of the production setup, as shown in Fig. 1 or as part of continuous improvement when better cutting tools are to be tested. Cutting speed, feed and depth of cut are optimized through trial and error and are mostly based on the expert knowledge of the machine operator and empirical rules in machine data handbooks [5]. If the tooling engineer has selected the right tools for the right material conditions, and the machine operator has selected the right machining parameters, the manufacturer prescribed tool life, as well as other desired outcomes, are achieved. Also, considering that tooling can account for up to 12 percent of the production tool life.

The studies so far take an experimental approach to get the best machining parameters to achieve the desired tool behavior in which various machining parameters are tried, and the parameters with the best tool life are adopted [7–9]. These studies treat machining parameter optimization as a one time job, but these machining parameters have to be optimized every time a new production line is being set up. The open-loop nature of the experimental approach presents a challenge of generalization; when the tool geometry, coating grades, or the workpiece material changes, the assumptions in the experimentation of the studies render the optimized parameter unusable. Adding to this, the optimized parameters don’t always work given the scholastic behavior of tool life. Therefore, it is better not to treat parameter optimization as a one time process.

**Fig. 2:**
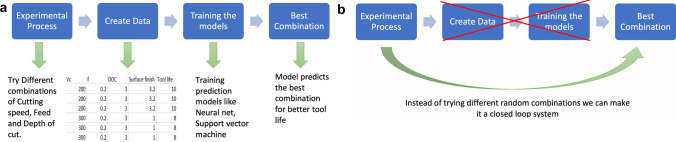
**a** Approach taken to get best machining parameters combination **b** The proposed closed loop system

Figure 2a gives a general overview of the approach taken by existing studies for parameter optimization. In the first step, an experiment is conducted with a different combination of cutting speed, feed, and depth of cut. A database is created with these experiments with target labels, which can be different depending on the objective. In the third step, the database is used to train prediction models or optimizers. Finally, the best combination of the machining parameters is predicted by the models. This approach works well as long as the tool behaves the same way in every application it is used. Figure 2b gives the proposed approach. In the proposed system, the database creation and training prediction models and optimizers are eliminated by making the experimentation process a closed-loop system. The closed-loop nature of the system has two advantages; one, instead of wasting time in trying different combinations, the closed nature tries the Very few studies have taken a closed-loop approach to parameter optimization. The existing closed-loop parameter controller depends on the passive signals like cutting force [10–12], spindle motor current [13], acoustic emissions signals [14]. Passive signals are good inputs to create closed-loop systems, but these systems require complex sensors close to the cutting action, which limits the size of the workstation. These systems are also prone to different noise in machine-shops. Most of the passive signal based systems do not consider other parameters that affect these signals like the length of the holder, chip breaker, condition, or rigidity of the machine while modeling the predictive algorithms; These systems work in a standard laboratory setup, but these parameters pose a challenge when these predictive models have to be scaled up to be implemented in the different industrial environments. The advantages of the online nature of these systems also can’t be utilized to a full extent because of the unidirectional nature of G-code execution used by most of the existing CNC machines [15–17], which don’t allow for real-time changes in G-codes. Vision-based systems, on the other hand, are not online systems, but they can work between cycles [18], and vision-based systems are more accurate than their indirect counterparts [18]. Inspired by the closed nature of the previously discussed studies and the problems experienced by the passive signal based systems while scaling up, combined with the ability of vision-based systems to address the shortcomings of these systems; the presented study proposes a closed-loop system alternative using a vision system.

**Fig. 3:**
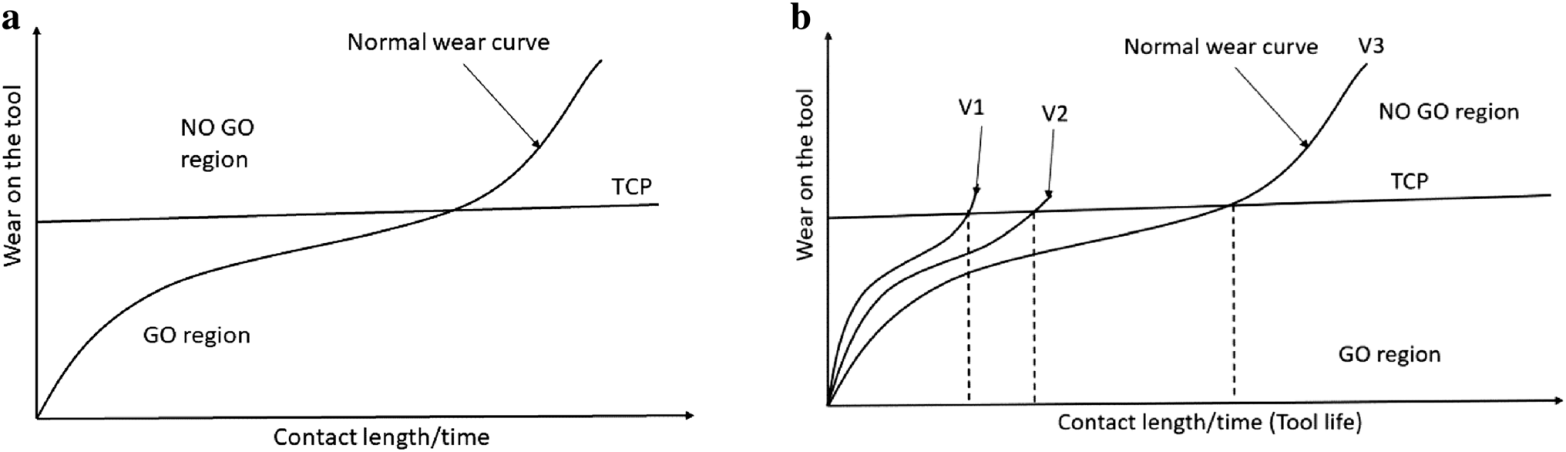
**a** Graphic illustration of normal wear curve for cutting tool with GO/ NO GO regions overlapped **b** Graphic illustration of wear curve for different cutting speeds and realtive change in TCP (adapted from [16])

The optimization systems with surface finish objective functions [19, 20], which form the bulk of the optimization studies, can be classified as quality-based optimization systems. But the drawback of current quality based systems is that they assume the quality requirement as a monolith, which is the surface finish, while as illustrated by Mamledesai et al. [21], there are different definitions for machining quality, and they are identified in tool change policies (TCP). It is a mammoth task to optimize these machining parameters for all quality requirements, which are also sometimes in conflict. Instead, we can encode the quality requirements as the useability of the tool, as suggested by Mamledesai et al. [21] the life of the tool can be divided into GO and NO GO region, where within the GO region, the tool is producing components that meet the design requirements which account for different quality requirements and enters the NO GO region when the tool starts to produce non-conforming parts. We can graphically superimpose these GO and NO GO regions on the normal wear curve [22, 23] as illustrated in Fig. 3a, where TCP is the threshold between GO and NO GO region. As seen in Fig. 3b, the quality is achieved through TCP irrespective of tool life, but when the normal wear curve (or desired wear mechanism) is achieved, TCP is pushed the furthest, and the TCP gets closer to zero when abnormal wear curves (or undesired wear mechanism) are seen. Therefore, there is a need for a parameter optimizer that will keep tool behavior closer to the normal wear curve and, as a consequence, also achieve better quality for more contact length or time.

The proposed system uses tool wear morphology as feedback for change in machining parameters. Tool manufacturers prescribe monitoring the wear morphology to optimize the machining parameters to achieve the best tool life and behavior [24–26]. There are different wear morphologies, and some of them are desired, and others are undesired morphologies. To achieve the maximum tool life (or normal wear curve), the desired wear morphology must prevail. The desired and undesired wear morphologies are discussed in Sect. 2. The decision based on the wear morphology also helps the tooling engineers generalize their knowledge across different tool geometry, coating grades, and working materials. The tooling engineers are taught to troubleshoot tool life using the wear morphologies and the remedy to those wear morphologies. As long as the tools have identifiable wear morphologies irrespective of the workpiece material, tool grade, or geometry, the respective remedy action can be taught; this helps the tooling engineers generalize their knowledge.

In this study, the authors take the approach a tooling engineer takes to optimize the machining parameters based on the visual information available. A tooling engineer first looks at the tool for the type of wear and approximates the level of wear. Based on this evidence, the engineer changes the parameter appropriate for the level of wear and type of wear. These appropriate changes are relative to the initial machining parameters, and the decision of the magnitude of changes in parameters is a skill developed over the years by the engineers.

The presented study replicates this decision-making through independent systems that first recognize the type of wear using the Artificial Neural Network (ANN) algorithm. The ANN algorithm is the best suited for this application as it is capable of autonomously detecting the region of interest and patterns in the images [27]. In the next step, the level of recognized wear is measured using commercially available measurement software [28]. Finally, the skills are captured in the system through fuzzy logic rules. The study utilizes fuzzy logic as it is flexible enough to capture a wide range of expert knowledge and converts them into linguistic variables that are defined by mathematical models [29–31]. Thus, performing the task of the tooling engineer independently. This independent decision-making system equips the new inexperienced machine operators with the preemptive remedy actions to achieve full utilization of the tool life. Also, the system can be used in fast-tracking of identification of the best machining parameters for new exotic materials. With the full integration of the proposed system with the machine controllers, the study can contribute to the concept of lights out machining without the need for human intervention.

The contribution of the study is a methodology that helps users determine the right combination of machining parameters and improve the tool life using fuzzy controller that replicates the human decision making partly when it comes to machining parameter optimization with tool life as the objective. The detection of wear morphology and measured values of the detected wear morphology serve as feedback to the controller, thus makes the proposed system a closed-loop system. This approach will also help in generalizing the system across different working materials, tool geometries, and tool grades once the entire family of tool wear is integrated as the decisions are based on visual evidence of wear morphology.

The study encompasses wear detection, wear measurement, and fuzzy controller, the interface between these three systems is manual and presents future scope to automate these interfaces. The boundary conditions used for the linguistic variables in the case study are imitated to the point of proving that the methodology works and there is a need to further investigate the boundary conditions for these linguistic variables. The study used a manual lathe which did not allow for independent control of feed rate therefore the remedy actions involving the feed rates are beyond the scope of the study. The study only considers flank wear, deformation, and built-up edge but there are other wear patterns like chipping, crater wear that warrant other remedial actions. These varieties of wear present an excellent opportunity to further investigate other remedy actions and their integration into the fuzzy control framework.

The rest of the paper is structured as follows. The background literature is discussed in Sect. 2. In Sect. 3, the methodology is proposed starting with the overview followed by the basic concepts used in the system. In Sect. 4, the case study and the results are demonstrated. Finally, the further development needs in the full implementation of the system and the conclusions are given in Sect. 5.

## Literature review

In this section, we first discuss the start of art technologies, followed by the different objectives for optimization and deficiencies of these systems. Finally, we explore the different prediction models used in the prediction.

Lan et al. [3] developed a system to maximize the tool life using fuzzy logic. This system optimizes the cutting speed, feed, and depth of cut using fuzzy rules, but there is no feedback loop in this system. Schultheiss et al. [32] studied the possibility of using a previously used tool for secondary machining operations. The authors in this study propose using alternatively left and right-hand cutters for using both sides of the nose. Haber et al. [12] developed a closed-loop fuzzy controller with an optimizer; this controller optimizes the feed rate based on force signals to achieve better tool life in drilling applications. The study also identified the need to tune fuzzy controllers using feedback. Bhushan [33] discussed the identification of significant combinations of critical machining parameters to achieve improvements in tool life and power consumption. The study takes an experimental approach to identify these significant combinations. Zhang et al. [34] Proposed an objective function to minimize the energy consumption; this objective function accounts for the stochastic nature of tool wear with other machining parameters. Finally, the authors arrive at the best combination to achieve better energy consumption. Shi et al. [35], in their study, established the relation between tool wear and power consumption. Ribeiro et al. [36], in their system, analyzed and optimized the machining parameters with the surface finish as the objective using an experimental approach. Moshat et al. [37] pointed to the popularity of Taguchi methodology when it comes to parameter optimization. The study proposed a hybrid Principle Component Analysis and Taguchi methodology to solve the optimization problem. Thepsonthi et al. [7] developed a multiobjective particle swarm optimization-based model for obtaining burr-free surface features along with the tool life. Yan et al. [20] proposed a multiobjective optimizer that considered production rate, cutting quality and energy consumption; the right cutting conditions are determined using weighted grey relational analysis. Ramesh et al. [19] investigated the optimal cutting parameters for better surface finish and better tool life using grey relation analysis and techniques for order preferences by similarity to ideal solution method. El-Hossainy et al. [38] introduced an optimizer using LINGO software; the software was optimizing different objective functions, and one of them is tool life. The system considered cutting speed, feed, and depth of cut as independent variables.

Surface finish [9, 39–43] is one of the primary objectives to optimize the machining parameters where inputs to prediction models are cutting speed, feed, and depth of cut, among others, and the model is expected to predict the surface finish. The drawbacks of using surface finish as an objective function are discussed in the previous section. The effects of various parameters on power consumption [42] are also studied extensively to provide the best working parameters that consume the least power. Often studies quote sustainable manufacturing while optimizing power consumption, but the studies do not consider the environmental impacts of the carbide ore extraction, supply chain, and how will improved tool life impact these aspects; there is a need for more holistic optimization when it comes to power consumption and sustainable manufacturing. Cycle time or production time [4] and Manufacturing cost [44, 45] are also a common objective to optimize the cutting parameters. Other than the above-mentioned objectives, some studies have also considered cutting force or load on machine [42] and material removal rate [46] as the objective to optimize the machining parameters. A complete review of different optimization objectives can be found in the study done by Rana et al. [47].

The machining parameter optimization objectives mentioned in the previous paragraphs are essential as they affect machining quality and production cost. On the other hand, if surface finish, power consumption, and production cost are considered without considering tool life, the manufacturers run the risk of underutilizing the tool or, in the worst-case, end up using the wrong tool, which drives up the tooling cost. Therefore, there is a need for a system that can optimize the tool life. In the context of optimized tool life, if the other desired outcomes like surface finish, production time, and cost are not achieved, the tool selection is wrong and has to be changed in consultation with tooling engineers.

The parameter optimization study has used a variety of methodologies to achieve the desired objectives. ANN is one of the new methodologies used in the last decade [42]; this methodology establishes the nonlinear relations between the input variables and target variables. The relation later helps in the prediction of outcomes of using individual machining parameter combinations. The genetic algorithm [48] is also a commonly used methodology based on the basic principle of selection of the best solution to the optimization problem. Experimentation, which involves trying different parameters and determining the best paraments of the lot, is also a common approach; the Taguchi method [9] is used to design these experiments. In the experimentation approach, which forms the basis of the above-mentioned methodologies, there is no room for a closed-loop system, which can adjust the parameters based on the online feedback from the change in a machining environment. The experimental approaches are at best useful to generate machining parameters data for catalogs; even for these applications, the experiment is trying different combinations without reliable feedback. The findings of these approaches are also limited to the material they are experimenting with or the tool geometries that are used in the studies. If the material or the tool geometry or the tool coating grade changes, the assumptions make the generalization of the findings for a different material or tool impossible. Therefore, there is a need for a methodology that can arrive at the best machining parameters using reliable feedback.

The proposed system develops a feedback loop and a closed system by optimizing the parameters based on the wear condition of the tools. The wear on the cutting tool is unavoidable. There are, however, desired and undesired wear patterns. The desired wear morphologies must prevail for the full utilization of cutting tools (or normal wear curve). Abrasion wear is the removal of small fragments [49] from the tool, which relatively preserves the rake angles of the cutting tool, giving the best life designed by the manufacturer. The abrasion wear pattern is also termed as normal flank wear by the tooling engineers. The other wear mechanism is plastic deformation, which significantly changes the working angles [49] of the insert rendering it unfit for machining in a short cutting time, this type of tool wear is commonly seen while machining high melting point material at high cutting speeds. The adhesive wear pattern is the other commonly seen wear pattern in the cutting insert, where the material being cut adheres to the cutting edge and the rake face [49], this leads to change in cutting angles and poses a risk to smooth chip flow which makes the tool unfit for machining, Built-Up Edge (BUE) is the industrially used term for this kind of wear pattern. The undesired wear patterns also lead to imperfections such as chatter marks, edge fettering, poor surface finish among others these effect the quality standards. The pictorial examples of these imperfections can be seen in the study conducted by Mamledesai et al. [21]. Considering that the plastic deformation and adhesive wear patterns drastically reduce the usability of the cutting tools (or generate abnormal wear curves), tool manufacturers prescribe remedy actions to achieve abrasive wear pattern, which is the ideal wear pattern to realize the full life of the cutting tool. The remedy actions to achieve abrasion wear patterns are discussed later in Sects. 3.3.

Parameter optimization based on tool condition monitoring can be done using indirect and direct monitoring methods [50]. Indirect methods use data from one or more of vibration [51–53], sound [54], and force [52] sensors. On the other hand, direct methods rely on first-hand evidence, like images of tools [55]. While indirect methods are online systems and give information on real-time bases, they are less accurate and susceptible to noise when the systems are deployed in machine shop floors [56]. Indirect systems are also trained for predictions based on specific experimental data provided by the sensors, and the model needs to be retrained if any of the parameters in the experiment change. For example, if the vibration sensor-based model is trained for finishing geometry, the same model can’t be used if the geometry changes to roughing geometry as the vibration levels are higher for roughing geometry; the same can be implied for other indirect methods. Direct systems like vision-based systems are not real-time systems but are in process systems; they can be designed to work in between cycles [18] and tool change programs. Since direct systems are based on first-hand evidence, they present the advantage of higher accuracy. Also, the vision systems can be placed away from the metal cutting; this allows them not to interfere with machining operations. That is why vision systems have gained popularity in inspects [57], collision detection [15, 16], and other applications. Direct systems can also be trained to monitor wear morphologies, which have specific remedial actions to achieve desired wear morphology. These remedy actions are common to different tool geometries, coating grates and workpiece materials. The ability to work with wear morphologies allows the system to generalize the remedy rules for different applications. Considering the higher accuracy of direct methods, combined with the ability to generalize remedy rules, the computer vision-based direct method is selected to create a feedback loop for a closed machining parameter optimization system that can respond to change in tool condition.

The gap in continuous machining parameter optimization using reliable feedback in the context of tool life is an area with scarce publications. As is evident in the literature discussed in the previous paragraphs, the proposed system is designed to address this gap. The developed system is a combination of a Convolutional Neural Network (CNN) and Fuzzy Logic (FL) methodology. The previous studies use FL for tool condition monitoring [58, 59], but FL is not the best approach for feature recognition since the feature descriptions have to be hardcoded in terms of fuzzy rules, which takes a considerable amount of computational memory and also FL systems can't accommodate new situations not bound by the rules [60]. In this regard, CNN approaches are more accurate and also don’t require the feature definition stage [61]; this expedites the training process and also improves the ability to recognize a variety of wear morphologies. FL, however, is efficient in converting human knowledge into variables computers can understand [60]. The FL in the proposed methodology is used to model the expert and tool manufacturer’s troubleshooting knowledge. The proposed hybrid system uses CNN as the feedback and FL as the controller, which selects and adapts the machining parameter.

## Hybrid Fuzzy controller with an image feedback system

**Fig. 4:**
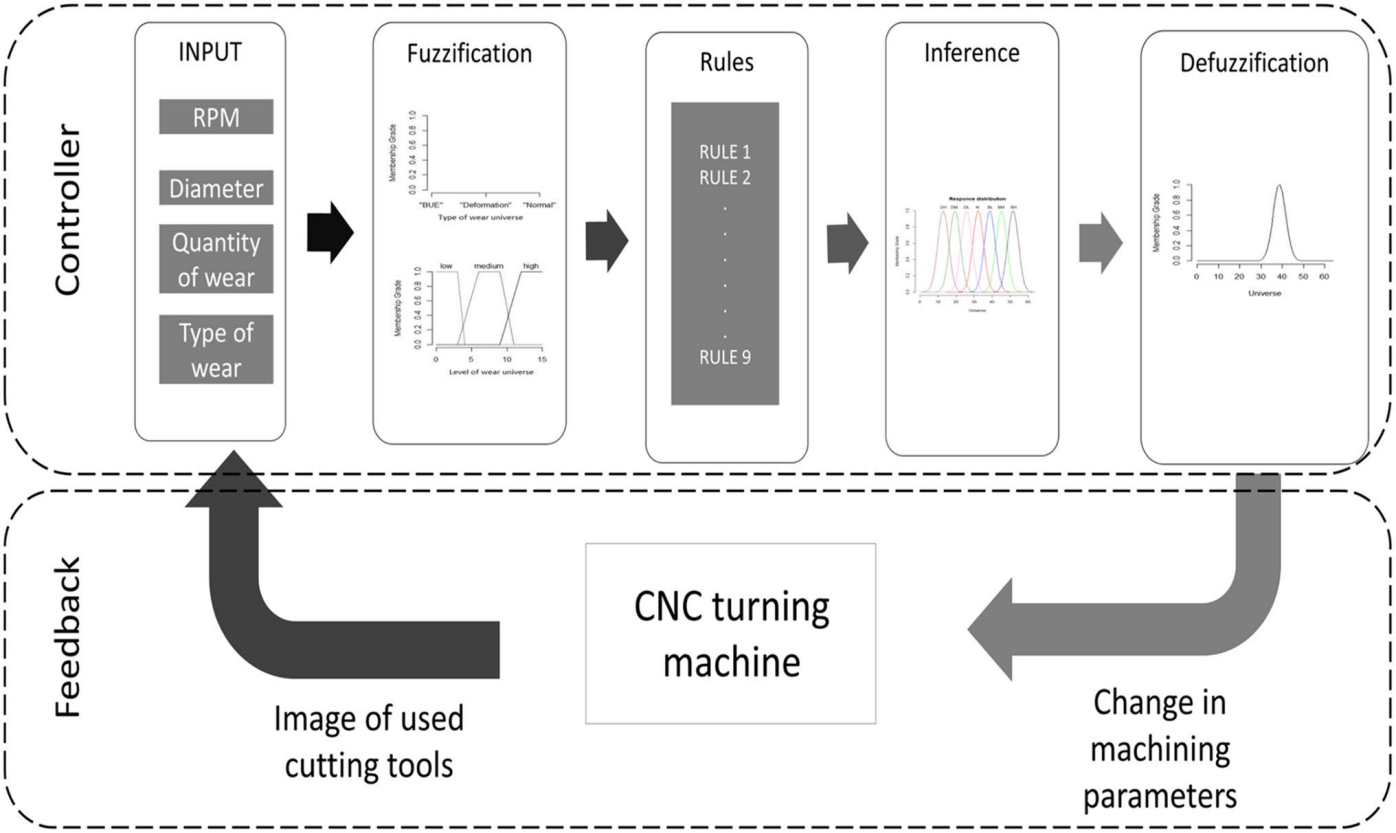
Overview of the fuzzy controller

The overview of the proposed fuzzy controller can be seen in Fig. 4. The proposed system is divided into the controller and the feedback sections. The feedback section consists of the wear classifiers that classify the type of wear on the tool and the approximates amount of wear on the tool. The type of wear, amount of wear, the component diameter, and spindle revolutions per minute (RPM) form the inputs to the controller; these are further elaborated in Sect. 3.1. The first step in the controller is the fuzzification, where the change in cutting speed, wear type, and lever of wear are converted to linguistic variables discussed in Sect. 3.2. In Sect. 3.3, the rule base, which forms the intelligence of the controller for remedial actions as suggested by tooling engineers and tool manufacturers, is developed. The output of the controller is a crisp number that is used to control the cutting speed of the machine. The output of the system is a remedial action to achieve the desired wear morphology that improves tool life. The process of relying on the evidence of wear morphology, amount of wear, and the initial machining parameters replicate the tooling engineer decision-making process when it comes to machining parameter optimization. The techniques of output inference and defuzzification are discussed in Sect. 3.4.

### Inputs to controller

The controller uses four inputs diameter of the component, RPM, type of wear, and level of wear. The cutting speed (${\mathrm{V}}_{\mathrm{c}}$) in meters per minute is calculated using Eq. 1 [62], where D is the diameter of the component to be machined in millimeters, and N is RPM of the workpiece.

1 $${\text{V}}_{{\text{c}}} = \left( {\pi {\text{DN}}} \right)/1000$$

There have been many studies in wear type identification and wear amount estimation fields. Sun and Yeh [63] developed an image processing methodology that can recognize the type of wear, and the level of wear is estimated by accounting for the number of pixels in the wear region. Wu et al. [61] took a neural network approach to identify the type of wear pattern and used a minimum circumscribed rectangle to get the quantity of the wear. The proposed system uses a neural network approach to identify the type of wear automatically by capturing the images of the used tools, and the amount of wear is manually calculated. However, there are other technologies developed that can also automate the amount of wear calculation.

Neural networks are one of the most used methods in image recognition. The neural network allows for automatic feature extraction by learning the nuanced differences in the images. The images are manually classified into different wear categories and are used to training and validate the classification models. Once the training is complete, the model can automatically identify the different wear patterns by uploading the new images.

The CNN architectures use different layers which perform different actions on the images. The convolution layers, narrow down on the region of interest and create useful descriptions of the images which make them best suited to work with images [64]. The output of the convolution layers then passes through the pooling layer, which in the case of the proposed architecture is a max-pooling layer which reports the maximum value in the predefined image pixel neighborhood. Max pooling layers make the proposed architecture more robust against small translation in image pixel data [65]. The dense layers are the fully connected layers where each neuron is interacting with all the neurons of the previous layers [66]. The last dense layer has the same amount of neurons as the number of wear classes, the output of the layer is the network’s prediction for the image belonging to three classes. This is summarized in Eq. 2 where ${\mathrm{y}}_{\mathrm{p}}$ is the prediction of the model.

2 $${\text{y}}_{{\text{p}}} = { }\left\{ {\begin{array}{*{20}c} 0 \\ 1 \\ 2 \\ \end{array} \begin{array}{*{20}c} {\text{if the image has BUE}} \\ {\text{if the image has deformation}} \\ {\text{if the image has normal wear}} \\ \end{array} } \right.$$

Activation functions are commonly used in the neural network to allow them to accommodate and learn non-linear functions [65], Rectified Linear Unit (ReLU) is commonly used in hidden layers of the network architectures as they return zero gradient value of negative nodes and the node value for positive inputs this improves the computation easy [67]. The softmax activation function is used in the final layer to represent probability distribution over different classes, which is a common practice in classifier architectures [65].

The parameters are where the intelligence of the layers are stored in terms of weights. These weights are fine-tuned by backpropagation in the training process. The model uses categorical cross-entropy as loss function [65] and ADAM as the optimizer for training and optimizing the weights [66]. More information about the training and optimization of neural network architectures can be found in [61, 65]–[68]. The proposed system uses the CNN architecture proposed in Table 1 to classify the wear type.

**Table 1:** CNN architecture for wear type classification model

	Layer type	Input shape	Output shape	Activation function	Parameters
1	Convolution layer	200,200,3	198,198,32	ReLU	896
2	Max pooling layer	198,198,32	99,99,32		0
3	Convolution layer	99,99,32	97,97,32	ReLU	9248
4	Max pooling layer	97,97,32	48,48,32		0
5	Convolution layer	48,48,32	46,46,64	ReLU	18,496
6	Max pooling layer	46,46,64	23,23,64		0
7	Convolution layer	23,23,64	21,21,64	ReLU	36,928
8	Max pooling layer	21,21,64	10,10,64		0
9	flatten (Flatten)	10,10,64	6400,1		0
10	Dense layer	6400,1	50,1	ReLU	320,050
11	Dense layer	50,1	35,1	ReLU	1785
12	Dense layer	35,1	10,1	ReLU	360
13	Dense layer	10,1	3,1	Softmax	33

The amount of wear is manually demarcated on the images of the used tools; although the magnitude can also be automatically generated by technologies discussed in [61, 63, 69], and many other studies, this work is not replicated. The type of wear (${\mathrm{y}}_{\mathrm{p}}$), amount of wear in terms of micrometers, and cutting speed (${\mathrm{V}}_{\mathrm{c}}$) form the inputs to the fuzzy controller.

### Fuzzification

There are two variables, type of wear $\chi_{T}$ and the amount of wear $\chi_{A}$ which form the input to the fuzzy systems. ${\mathcal{L}}_{{\text{T}}} { = }\left\{ {{\text{"BUE"}}, {\text{"Deformation"}}, {\text{"Normal"}} {\text{"wear"}}} \right\}$, and ${\mathcal{L}}_{{\text{A}}} { = }\left\{ {{\text{"Low"}}, {\text{"Medium"}}, {\text{"High"}}} \right\}$ are the family of linguistic values for the type of wear and amount of wear, respectively. L_T_ is the label used from family ${\mathcal{L}}_{{\text{T}}}$ and L_A_ is the label used from family ${\mathcal{L}}_{A}$ this is summarized by Eqs. 3 and 4.

3 $${\text{L}}_{{\text{T}}} = { }\left\{ {\begin{array}{*{20}c} {{\text{BUE}}} \\ {{\text{Deformation}}} \\ {{\text{Normal}}\;{\text{wear}}} \\ \end{array} } \right.$$

4 $${\text{L}}_{{\text{A}}} = { }\left\{ {\begin{array}{*{20}c} {{\text{Low}}} \\ {{\text{Medium}}} \\ {{\text{High}}} \\ \end{array} } \right.$$

The amount of wear has a trapezoidal membership function [70]; this is summarized in Eq. 5, where x_a_ is the measured value of wear on the cutting tool in micrometers and p,q,r,s are the boundary values of the membership function. Similarly, for the type of wear, the membership function is singleton given in Eq. 6, where ${\mathrm{x}}_{0}= {\mathrm{y}}_{\mathrm{p}}$.

5 $${\upmu }_{{\text{A}}} \left( {{\text{x}}_{{\text{a}}} ;{\text{ p}},{\text{q}},{\text{r}},{\text{s}}} \right) = \left\{ {\begin{array}{*{20}c} 0 \\ {\begin{array}{*{20}c} {\left( {{\text{x}} - {\text{p}}} \right)/\left( {{\text{q}} - {\text{p}}} \right)} \\ {\begin{array}{*{20}c} 1 \\ {\begin{array}{*{20}c} {\left( {s - {\text{x}}} \right)/\left( {{\text{s}} - {\text{r}}} \right)} \\ 0 \\ \end{array} } \\ \end{array} } \\ \end{array} } \\ \end{array} \user2{ }\begin{array}{*{20}c} {\begin{array}{*{20}c} {\begin{array}{*{20}c} {{\text{x}}_{{\text{a}}} \le {\text{p}}} \\ {{\text{p}} < {\text{x}}_{{\text{a}}} { } \le {\text{q}}} \\ \end{array} } \\ {{\text{q }} < {\text{x}}_{{\text{a}}} { } \le {\text{r}}} \\ \end{array} } \\ {{\text{r}} < {\text{x}}_{{\text{a}}} { } \le {\text{s}}} \\ {{\text{x}}_{{\text{a}}} > {\text{s}}} \\ \end{array} } \right.$$

6 $${\upmu }_{{\text{T}}} \left( {{\text{x}}_{{\text{T}}} ;{\text{x}}_{0} { }} \right) = {\text{ f}}\left( {\text{x}} \right) = \left\{ {\begin{array}{*{20}c} 1 \\ 0 \\ \end{array} \begin{array}{*{20}c} {{\text{x}}_{{\text{T}}} = {\text{x}}_{0} } \\ {{\text{x}}_{{\text{T}}} \ne {\text{x}}_{0} } \\ \end{array} } \right.$$

The response ($\mathfrak{R}$) is divided into seven linguistic variables $\mathcal{R}$. Where, $\mathcal{R}$ = {Deformation High (DH), Deformation Medium (DM), Deformation Low (DL), Normal (N), BUE High (BH), BUE Medium (BM), BUE Low (BL)}. R is the label used from family $\mathcal{R}$. The Gaussian membership function [70] for the response variable (${\upmu }_{\mathrm{R}}$) is given in Eq. 7, where c is the mean of the distribution, s is the standard deviation, and y is the output value. The mean of the linguistic response variables is dependent on the initial cutting speeds. The Gaussian membership is carefully chosen because we can easily control the distribution with two parameters compared to four in the trapezoidal membership function.

7 $${\upmu }_{{\text{R }}} \left( {{\text{y}};{\text{c}},{\text{ s}}} \right) = {\text{ e}}^{{\left( { - { }\frac{{\left( {{\text{y}} - {\text{c}}} \right)^{2} }}{{2{\text{s}}^{2} }}} \right)}}$$

### Rules

The rules for the fuzzy controller are developed using the knowledge base of troubleshooting guides from different tool manufacturers. The different statements extracted from troubleshooting guides are given in Table 2. The troubleshooting guides only suggest the overall remedy actions, but the magnitude of change in cutting speed or the feed rate is the skills developed by tooling engineers over time and experience; these skills are captured in the fuzzy rules.

**Table 2:** Remedy actions from knowledge base troubleshooting guides published by tool manufacturers

Wear mechanism detection	Remedy statement for cutting speed	Reference	Remedy statement for feed rate	Reference
BUE	Increase cutting speed	[24, 25, 71]		
Normal wear		Desired wear pattern	[26, 71, 72]	
Deformation	Decrease cutting speed	[24, 25, 66]	Decrease feed rate	[24, 25]

Based on the information from the knowledge base and the tooling engineer’s skills, the fuzzy rules (${\mathcal{H}}^{i}$) are developed, the basic fuzzy rule is given by Eq. 8. The different linguistic values of L_T_, L_A,_ and R for rule i are summarized in Table 3. The fuzzy rules model the expert statement; for example, rule 1 states that if the wear type is “BUE” and wear amount is “High” then increase the cutting speed by “BH,” where “BH” can be a percentage increase from initial cutting speed.

8 $${\mathcal{H}}^{{\text{i}}} { = } \left\{ {{\text{IF}}{\mkern 1mu} \;\chi_{{\text{T}}} {\mkern 1mu} \;{\text{is}}\;{\mkern 1mu} {\text{L}}_{{\text{T}}} {\mkern 1mu} \;{\text{AND}}\;{\mkern 1mu} \chi_{{\text{A}}} \;{\mkern 1mu} {\text{is}}{\mkern 1mu} \;{\text{L}}_{{\text{A}}} {\mkern 1mu} \;{\text{THEN}}{\mkern 1mu} \;\Re \;{\mkern 1mu} {\text{is}}\;{\mkern 1mu} {\text{R}}} \right\}_{{\text{i = 1}}}^{{9}}$$

**Table 3:** Linguistic variables for different fuzzy rules

i	L_T_	L_A_	R
1	BUE	High	BH
2	BUE	Medium	BM
3	BUE	Low	BL
4	Normal	High	N
5	Normal	Medium	N
6	Normal	Low	N
7	Deformation	Low	DL
8	Deformation	Medium	DM
9	Deformation	High	DH

### Inference and defuzzification

Mamdani-Assilan fuzzy inference method is used for fuzzy inference. This method is suitable for the application at hand as it can work with the conjunctive interpretation of fuzzy rules in the canonical form given in Eq. 8 [70]. The conjunctive “AND” is interpreted as the minimum ($\wedge$) [70]. The inference results from each rule are finally added using maximum ($\vee$) operation [70]. The final inference value ${\upmu }_{{{\text{R}}^{*} }} \left( {\text{y}} \right)$, which gives the area under all the triggered rules is given in Eq. 9.

9 $${\upmu }_{{{\text{R}}^{*} }} \left( {\text{y}} \right) = { } \vee_{{{\text{i}} = 1}}^{9} \left[ {{ }\begin{array}{*{20}c} {{\upmu }_{{\text{T}}}^{{\text{i}}} \left( {{\text{x}}_{{\text{T}}} } \right)} & \wedge & {\begin{array}{*{20}c} {{\upmu }_{{\text{A}}}^{{\text{i}}} \left( {{\text{x}}_{{\text{A}}} } \right)} & {\begin{array}{*{20}c} \wedge & {{\upmu }_{{\text{R}}}^{{\text{i}}} \left( {\text{y}} \right)} \\ \end{array} } \\ \end{array} } \\ \end{array} }\right]{\text{ y}} \to _{ } {\mathbb{Y}}$$

The defuzzification is done using the center of gravity (COG) method [70] where the crisp number for the new cutting speed ${\text{y}}_{{{\text{new}}}}$ is returned by the controller. The COG of the aggregate area of all the rules represented by Eq. 9 is calculated using Eq. 10. ${\text{y}}_{{{\text{new}}}}$ is the new cutting speed used for the new machining cycle, which is influenced by initial cutting speed, type of wear, and amount of wear detected on the tool used in the previous cycle.

10 $${\text{y}}_{{{\text{new}}}} = \frac{{\mathop \smallint \nolimits_{{\mathbb{Y}}}^{ } \begin{array}{*{20}c} {\text{y}} & {{\upmu }_{{{\text{R}}^{*} }} \left( {\text{y}} \right)} & {{\text{dy}}} \\ \end{array} }}{{\mathop \smallint \nolimits_{{\mathbb{Y}}}^{ } \begin{array}{*{20}c} {{\upmu }_{{{\text{R}}^{*} }} \left( {\text{y}} \right)} & {{\text{dy}}} \\ \end{array} }}$$

The fuzzy controller developed can only work with the cutting speed. Similarly, the fuzzy controllers can be developed for other machining parameters like feed rate and depth of cut. Cutting speed was considered as there is a consensus among the previous studies that the cutting speed is one of the most influential factors when it comes to tool life [73]^74^.

## Case study

The purpose of this case study is to demonstrate the ability of the hybrid system to take remedy actions based on the wear morphology detected from the images of the cutting tools, and to demonstrate the positive effects of those remedy actions. Proving the effectiveness of the magnitude of changes (response) and the limits of linguistic variables is beyond the scope of the case study, and there is a need for more research in this direction.

**Fig. 5:**
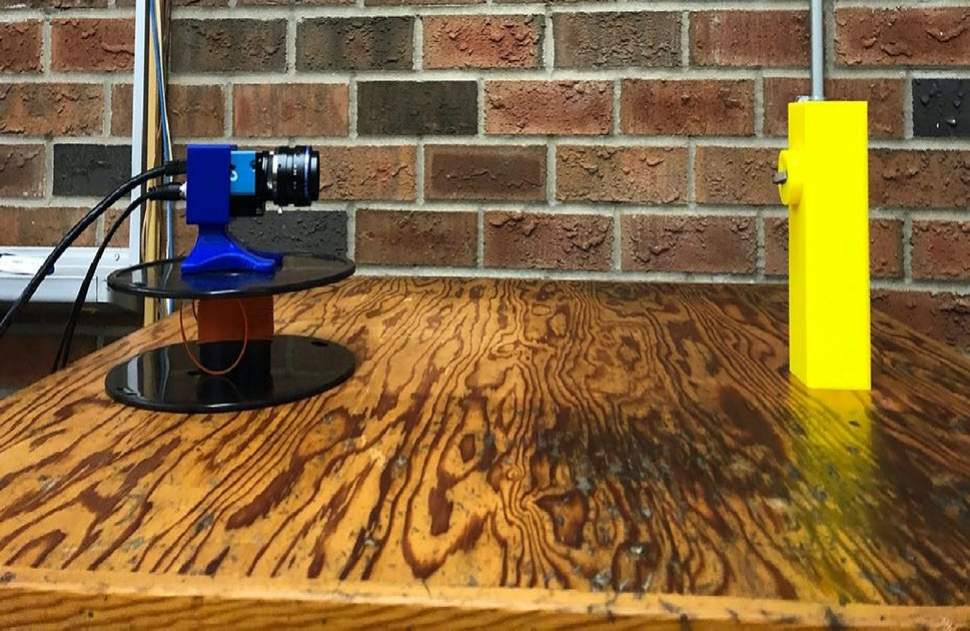
Image capturing setup

**Fig. 6:**
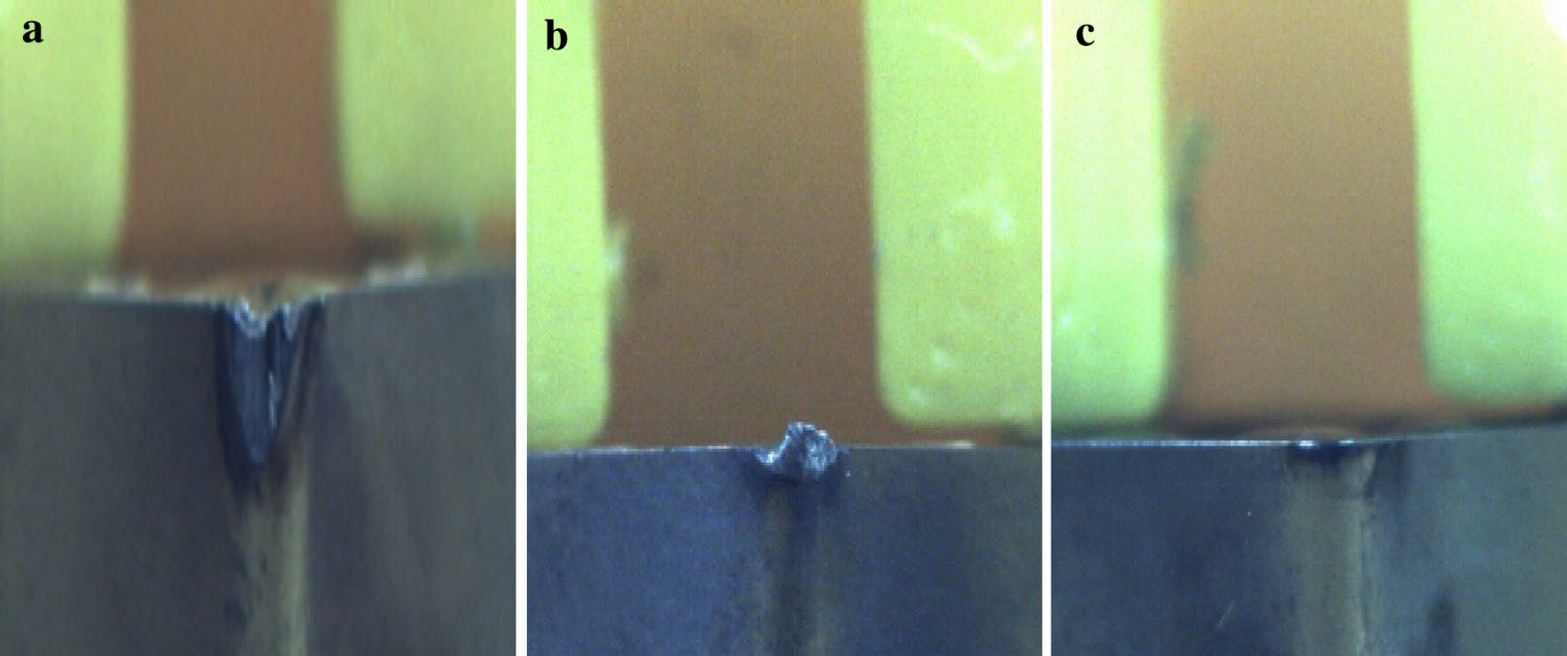
Examples of 200 × 200 pixels image for a** (a)** Deformation,** (b)** BUE and** (c)** Normal wear

The case study started with the training and deployment of wear classification CNN. For the training, first, the images of used TNMG, CNMG and uncoated High Speed steel cutting tools that have BUE, deformation, and normal wear patterns are acquired using a GigE DFK 33GP006 image sensor with TCL 3520 5MP lens with a 35 mm focal length; the setup can be seen in Fig. 5. The examples of images from different categories can be seen in Fig. 6. The image sensor has a resolution of 2592 * 1955. The neural network models were built and trained in the Intel Core i5 processor using the Tensorflow backend and Keras higher level package. For the wear classification model, a total of 207 images were used to train the model discussed in Table 1, and 89 images were used for validation of the model. The images, when captured, were of different sizes but were resized to 200*200*3 RGB images using the EBImage [75] package.

**Fig. 7:**
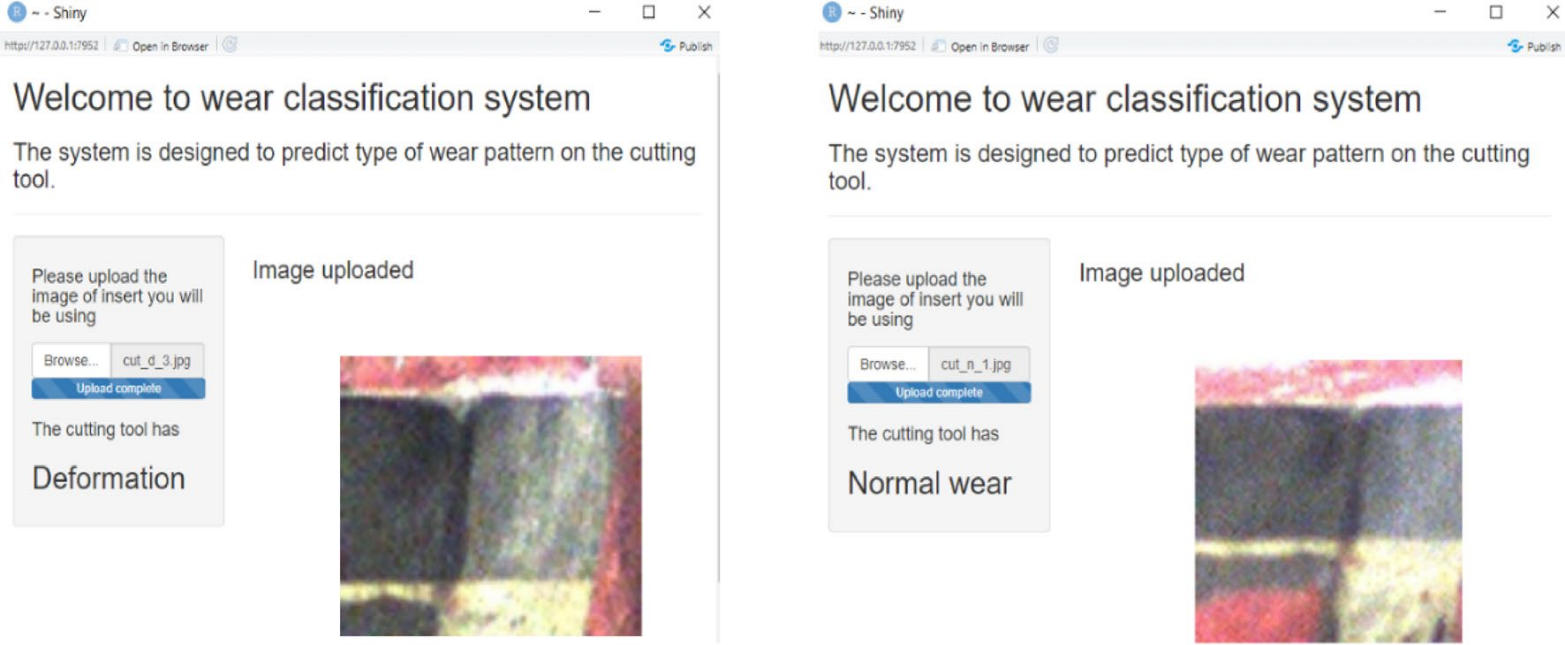
GUI for the wear classification model

The confusion matrix of wear type classification model’s predictions on the validation data set is shown in Table 4. The model has 86.52 percent accuracy and 0.3752 loss on the validation data set. The confusion matrix illustrates that the model performed reasonably well in identifying the wear patterns; the numbers in the diagonal of Table 4 are the correct predictions.

**Table 4:** Base model confusion matrix

Prediction label	Actual label		
	BUE	Normal	Deform
BUE	20	2	0
NORMAL	3	27	1
DEFORM	1	5	30

The wear classification model is then deployed using a Graphical user interface (GUI). The GUI asks the user to upload the image of the used tool, and the output is the type of wear, this is manually fed to the fuzzy controller. The examples of the deployed GUI can be seen in Fig. 7.

**Fig. 8:**
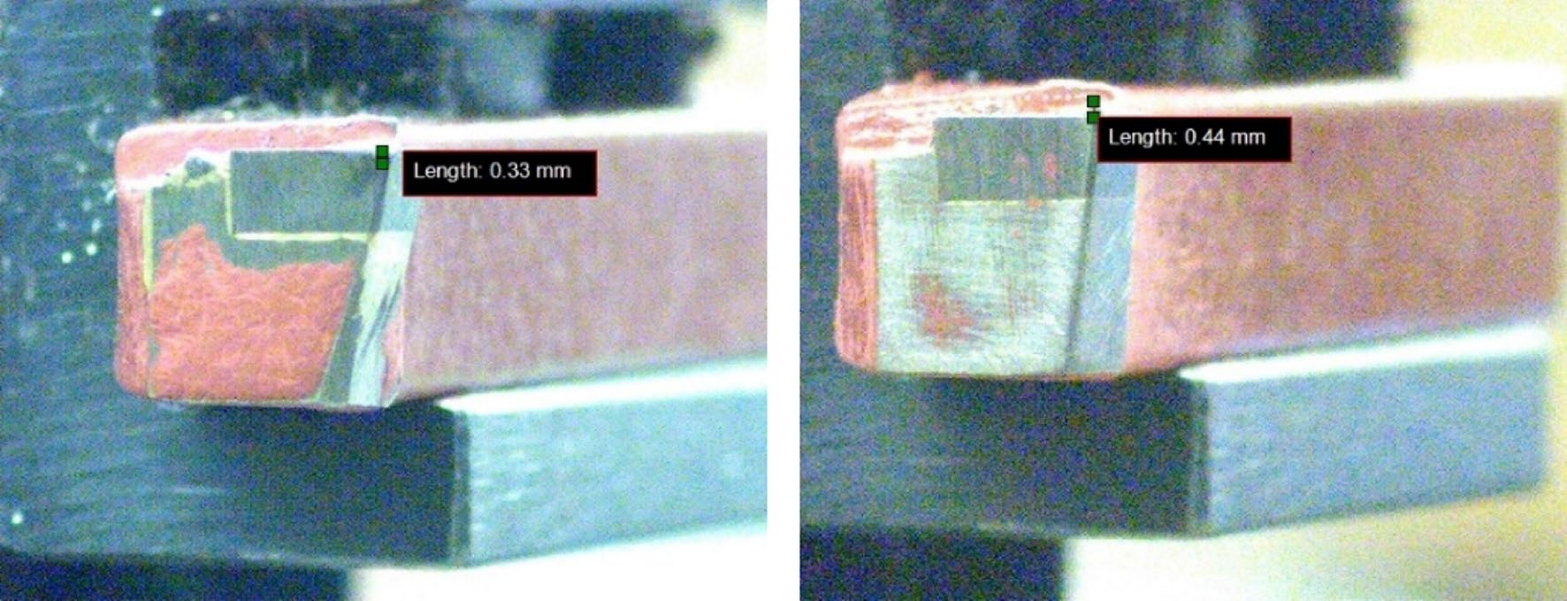
Amount of wear measurement using IC Measure software

The amount of wear can be automatically measured using various technologies discussed in Sect. 3.1; however, in the proposed system, the measurement is done manually using commercially available IC Measure software [28]. The software uses image processing techniques. The calibration process prescribed by the makers were followed before measurement. Since the software is the intellectual property of the company further details are not shared by the makers. The examples of the measurements can be seen in Fig. 8.

**Fig. 9:**
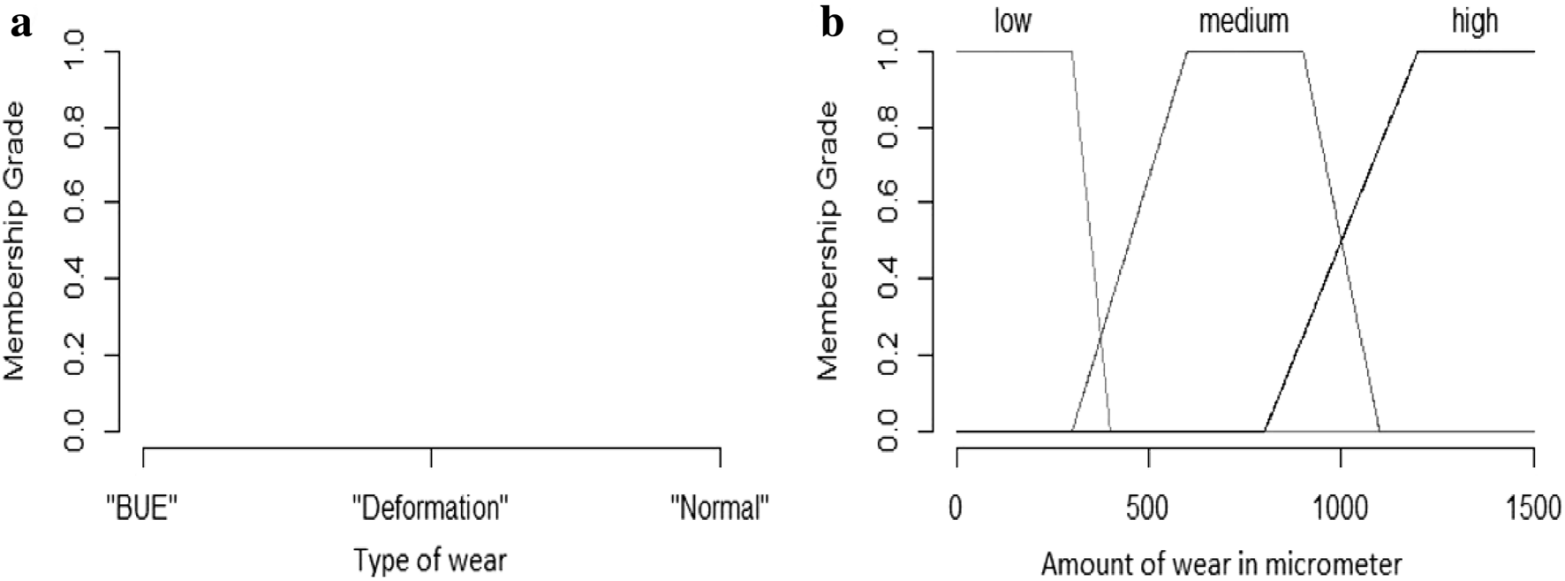
Linguistic input variables a) type of wear b) amount of wear

The boundary values used to define the amount of wear trapezoidal membership function (p,q,r,s) are summarized in Table 5. These values are used with Eq. 5 to generate the membership functions for high, medium, and low linguistic variables as shown in Fig. 9b; similarly, the membership function for the type of wear is shown in Fig. 9a. The c and s values used to define the response linguistic variable’s Gaussian membership functions are summarized in Table 6.

**Table 5:** Boundary points for wear amount membership functions

${A}_{x}$	p	q	r	S
Low	10,000	200	300	400
Medium	350	600	900	1100
High	900	1200	1500	10,000

**Table 6:** Mean and standard deviation values for different response linguistic variables

R	c	s
BH	V_c_ + (0.6 V_c_)	3
BM	V_c_ + (0.4 V_c_)	3
BL	V_c_ + (0.2 V_c_)	3
N	V_c_	3

R	c	s
DM	V_c_−(0.4 V_c_)	3
DH	V_c_−(0.6 V_c_)	3
DL	V_c_−(0.2 V_c_)	3

For the fuzzy controller evaluation, Micro-Mark mini-lathe 7 × 16 is used for machining. The tools used are uncoated high-speed steel tools. The workpiece material is Stainless steel 304. The cutting speed was monitored by recording the diameter of the component and the RPM (measured using REED instruments R7050 photo tachometer and counter). The standard operating procedure in Table 7 was followed for collecting the data; the steps are repeated after every cut of 78 mm.

**Table 7:** Standard operating procedure for collecting data

*Step 1:* Start the rotation and set the RPM to a predetermined level, as indicated by the fuzzy controller
*Step 2:* Carry out the metal cutting using automatic leadscrew feed
*Step 3:* Capture the image of the used tool and record the wear detected by wear classification GUI
*Step 4:* Measure the wear on the tool using IC Measure software if the wear is BUE or Deformation
*Step 5:* Record the diameter of the workpiece after the machining
*Step 6:* Input the diameter, RPM, type of wear, and measured wear to the fuzzy controller
*Step 7:* Record the cut number, cutting speed, and RPM suggested by the fuzzy controller

**Fig. 10:**
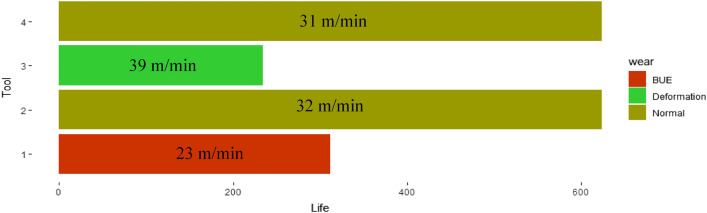
Consolidated results from the experimental data presented in Appendix 1

The data is collected for four cutting edges; the result of the experiment is shown in Appendix 1 and summarized in Fig. 10. Tool 1 and Tool 3 are initiated with abnormally low (23 m/min) and high (39 m/min) cutting speeds, respectively, which generated BUE and Deformation. The use of tools is stopped when the undesired wear patterns are detected. The life for Tool 1 and Tool 2 in terms of contract length is 312 mm and 234 mm, respectively. When the undesired wear patterns are detected, the fuzzy controller suggested the change in cutting speed; when the suggestion is used while machining with Tool 2 and Tool 4, the tool life improved by more than 100 percent, as shown in Fig. 10.

The study illustrates the ability of the system to detect different wear morphologies and take remedy actions by changing the cutting speed to achieve the desired wear patterns and, in this process, achieve better tool life. The system can work with different materials and tool geometries as the remedy actions are based on the wear morphologies. The system was evaluated on a manual lathe, which did not allow for the control of feed rate.

The study considered cutting speed as the optimizing parameter as it has maximum impact on the tool life [73], 74]. To further advance the scope of this study the fuzzy controller will have to modified to be a multiple output optimization fuzzy controller as discussed in the study done by Rodic [76]. Similar fuzzy rules, as discussed in Sect. 3.3, will have to be developed for remedy actions that involve controlling feed rates and depth of cut based on the remedial actions prescribed for different wear patterns. The future work will be bidirectional. One, towards including a wider range of undesired wear patterns other than BUE and Deformation like chipping, crater wear, among others, which require us to control other machining parameters like feed rate and depth pf cut as part of remedy actions. Second, There is also a need to make the system completely automatic by integrating the outputs of the wear classification model, wear amount measurement tool with the input to the controller, this process in the proposed study is done manually.

## Conclusion

Machining parameter optimization is one of the extensively studied fields of manufacturing, with the objective of optimization being different. The proposed system uses the theory of wear mechanism to optimize the machining parameters. The objective of the study is to get the desired wear pattern when undesired wear patterns are detected to achieve better tool life. This problem is divided into two sections first, the detection of wear mechanism and level of wear, and in the second section, these detections are used as signals to trigger fuzzy rules, which change the machining parameter to obtain the desired wear pattern in the next cutting edge. The system uses CNN for the detection of wear mechanisms. The fuzzy controller uses the output of wear classifier, amount of wear, and current state of machining parameters as input to suggest changes to the machining parameters for the next cutting edge. The case study developed illustrates that when the suggested changes are incorporated, the tool life can be improved by 100 percent. Since the system is dependent on the wear morphology as feedback to the deployed parameters, the system is not limited by the working material or tool geometries.

## Appendix 1

See Appendix Table

**Table 8:** Hybrid fuzzy controlling experimental data

Input to the fuzzy controller						Output of the fuzzy controller			
Cut	Diameter	rpm	GUI output	V_c_	New diameter	Amount of wear	New V_c_	New rpm	Length mm
TOOL 1									
1	28.11	260	Normal	23	27.93	0	23	262	78
2	27.93	262	Normal	23	27.55	0	23	266	156
3	27.55	266	Normal	23	27.21	0	23	269	234
4	27.21	269	BUE	23	26.73	0.44	32	383	312
TOOL 2									
1	26.73	383	Normal	32	26.37	0	32	388	78
2	26.37	388	Normal	32	26	0	32	394	156
3	26	394	Normal	32	25.82	0	32	397	234
4	25.82	397	Normal	32	25.23	0	32	406	312
5	25.23	406	Normal	32	25	0	32	410	390
6	25	410	Normal	32	24.6	0	32	417	468
7	24.6	417	Normal	32	24.32	0	32	422	546
8	24.32	422	Normal	32	23.98	0	32	428	624
TOOL 3									
1	24.6	500	Normal	39	24.46	0	39	503	78
2	24.46	503	Normal	39	23.8	0	39	517	156
3	23.8	517	Deformation	39	28.53	0.33	31	345	234
TOOL 4									
1	28.53	345	Normal	31	28.14	0	31	350	78
2	28.14	350	Normal	31	27.75	0	31	355	156
3	27.75	355	Normal	31	27.41	0	31	359	234
4	27.41	359	Normal	31	26.92	0	31	366	312
5	26.92	366	Normal	31	26.53	0	31	371	390
6	26.53	371	Normal	31	26.24	0	31	375	468
7	26.24	375	Normal	31	25.8	0	31	381	546
8	25.8	381	Normal	31	25.45	0	31	386	624

^8^
